# Expanded range of eight orchid bee species (Hymenoptera, Apidae, Euglossini) in Costa Rica

**DOI:** 10.3897/BDJ.10.e81220

**Published:** 2022-07-28

**Authors:** Elise McDonald, Jacob Podesta, Christine Cairns Fortuin, Kamal JK Gandhi

**Affiliations:** 1 University of Georgia, D.B. Warnell School of Forestry and Natural Resources, Athens, GA, United States of America University of Georgia, D.B. Warnell School of Forestry and Natural Resources Athens, GA United States of America; 2 University of York, York, United Kingdom University of York York United Kingdom

**Keywords:** Costa Rica, premontane wet forest, Monteverde, orchid bees

## Abstract

**Background:**

The Monteverde region of Costa Rica is a hotspot of endemism and biodiversity. The region is, however, disturbed by human activities such as agriculture and urbanisation. This study provides a list of orchid bees (Hymenoptera: Euglossini) compiled from field surveys conducted during January-October 2019 in the premontane wet forest of San Luis, Monteverde, Costa Rica. We collected 36 species of Euglossine bees across four genera. We provide new geographic distribution and elevation data for eight species in two genera. Due to their critical role in the pollination of orchids and other plants, the distribution and abundance of Euglossine bees has relevance to plant biodiversity and conservation efforts. This is especially important in a region with a high diversity of difficult-to-study epiphytic orchids, such as in the Monteverde region.

**New information:**

A total of 2,742 Euglossine male individuals across four genera (*Eufriesea*, *Eulaema*, *Euglossa* and *Exaerete*) were collected in this study. Updated geographic distributions and elevation ranges were established for eight species of Euglossini in two genera: *Eufrieseamussitans* (Fabricius, 1787), *Eufriesearufocauda* (Kimsey, 1977), *Euglossadodsoni* (Moure, 1965), *Euglossadressleri* (Moure, 1968), *Euglossahansoni* (Moure, 1965), *Euglossaignita* (Smith, 1874), *Euglossatridentata* (Moure, 1970) and *Euglossaturbinifex* (Dressler, 1978). These are the first recorded occurrences of these species in the Monteverde region of Costa Rica, according to the Global Biodiversity Information Facility (GBIF) database (https://doi.org/10.15468/9f9kgp). This study also established expanded elevation ranges for *Euglossaallosticta*, *Euglossabursigera*, *Euglossamixta*, *Euglossaheterosticta* and *Euglossamaculabris*, though these five species have been previously recorded in the Monteverde region and, thus, are not described in detail here. Additionally, our capture of 123 *Eufrieseaconcava* individuals is significant, as it indicates its abundance in this region. Prior to this study, there was a single record of *E.concava* in the Monteverde region, documented in 1993.

## Introduction

Hymenoptera in the tribe Euglossini (orchid bees) are a diverse group of New World tropical pollinators accounting for up to 25% of total bee communities in the Neotropics (Roubik and Hanson 2004, McCravy 2016). Orchid bees have a geographical range spanning from Mexico to Argentina, with one species known in the southern United States. There are 268 documented species with 66 known to inhabit Costa Rica from four genera: *Eufriesea*, *Euglossa*, *Eulaema* and *Exaerete*. Orchid bees play a vital role in the pollination of orchids (Orchidaceae) and other plant groups (Dressler 1982, Botsch et al. 2017). Euglossine bees fly distances of up to 23 km per day and, thus, are important pollinators of pRoubik and Hanson 2004, [Bibr B7595043][Bibr B7595043], Roubik and Hanson 2004lants with low population densities, such as many orchid species (Janzen 1971, Tonhasca Jr et al. 2003, Wikelski et al. 2010). Nearly 700 orchid species rely exclusively on pollination by male orchid bees (Roubik and Hanson 2004). The male bees visit orchids to collect odorous compounds to attract females. The chemicals are stored in specialised setae-lined organs on the hind tibia of the males, a physical characteristic found nowhere else in the insect world (Eltz et al. 2005). Female orchid bees are polylectic and will collect pollen from a wide diversity of plant species in tropical forests to provision their offspring and, thus, contribute to reproductive success of many tropical plant species (Ferreira-Caliman et al. 2018).

The global decline of bees has been widely observed and is especially pronounced in areas of intense agriculture, urbanisation and pesticide use (Biesmeijer et al. 2006, Potts et al. 2010, Goulson et al. 2015, Koh et al. 2016, Sánchez-Bayo and Wyckhuys 2019). Though documented population trends of tropical bees are consistent with these global trends, studies on bees in the tropics are vastly under-represented and their status is largely unknown (Prado et al. 2017). The few long-term studies that exist document Euglossine population declines to varying degrees (Vega-Hidalgo et al. 2020a). Populations of some other wild bee groups have appeared largely stable in protected areas, such as nature preserves (Frankie et al. 1998, Cairns et al. 2005, Roubik and Villanueva-Gutierrez 2009, Archer 2013, Herrera 2019); however, declines in Euglossine populations have been recorded even in natural areas that have not been modified (Nemésio 2013, Nemésio et al. 2016, Hallmann et al. 2017, Storck-Tonon and Peres 2017, Rada et al. 2019, Vega-Hidalgo et al. 2020b). More multi-year, year-round studies are needed to fully elucidate the status of Euglossine and other tropical bees.

Euglossini vary in abundance with season, with some species (e.g. many *Eufriesea* spp.) only being active for a few months of the year (Nemésio 2011). In the lowland forests of Costa Rica, Euglossine bees exhibit seasonal variation in overall abundance and species diversity (Janzen et al. 1982). Hence, sampling throughout the year during the wet and dry seasons is necessary to fully assess the species diversity of orchid bees in an area. Another study examined fragmentation of habitat types at elevations of 900-1,400 m in southern Costa Rica (Brosi 2009). We sampled orchid bees in Monteverde in Costa Rica using baited traps. Most tropical bee studies are conducted in the understorey due to dense forests using pan traps and timed observations, making our study unique in both the sampling of the canopy and the trap type.

## General description

### Purpose

Euglossine bees were sampled in the Monteverde region of Costa Rica at an elevation immediately below the cloud forest (1,100-1,170 m above sea level), in what is considered premontane wet forest. To our knowledge, this area has not been previously sampled for orchid bees. This area was chosen as it provided the opportunity to sample understorey, canopy and open areas to capture a range of diversity in the orchid bee community. In this way, it is more representative of the general state of orchid bee diversity in the region; much of Central America's forests are degraded, with secondary forests and the remaining primary forests predominantly in reserves, in a patchwork of human-managed and semi-natural environments (personal communication, Jacob Podesta). It is, therefore, necessary to understand how Euglossine diversity responds to the anthropogenic changes in their geographic range to provide information for management strategies to promote and maintain orchid bee diversity. We collected a total of 2,742 specimens, represented by 36 species across four genera, all of which were adult males. We provide expanded geographic distribution and elevation ranges for eight bee species.

## Sampling methods

### Study extent

Three areas of the 62.7 hectare University of Georgia Costa Rica campus were sampled: Forest A (trap locations F1-F5), Forest B (F6-F10) and the open area through the developed part of campus (O1-O10) (Table 1). At each forest trap location, one trap was placed in the understorey and the canopy, so that Forests A and B each contained 10 traps. Sampling occurred for five consecutive days each month January-October 2019. As summarised in Table 2 and detailed below, Forest A was sampled January-October, Forest B was sampled in April and October and the open area was sampled in June-October.

### Sampling description

Male Euglossine bees are attracted to baited traps using artificial compounds that mimic those of desired orchids, a method employed regularly by researchers (Sandino 2004, Velez and Pulido-Barrios 2005, Hedström et al. 2006). Cineole is used in this study as it is considered a universal attractant for male orchid bees (Buchmann 2019). Traps used to sample bees were made using a 3.5 l container with lid, with an opening of 4 cm located on one side of the container. A single metal screw hook was attached to the top of the container lid and used to secure the trap. On the inside of the lid, a galvanised wire was used to secure a cotton ball wrapped in wire mesh which served as the attractant site for the cineole.

The dry season is typically marked by high wind speeds and little rainfall. Dry season sampling occurred January to May 2019. During this time, the five sites and ten traps in Forest A were sampled each month, with Forest B being sampled during the peak dry season in April.

The wet season sampling took place June to October 2019. In addition to the ten traps in Forest A, ten trap sites in the open area were added during the wet season. In October, the peak of wet season, Forest B was added, for a total of 30 operational traps. The Forest B site was sampled during the historical peak dry and wet seasons to provide additional data for comparison. We selected trapping sites where the elevation range along a transect was no more than 100 m.

**Figure 1.**
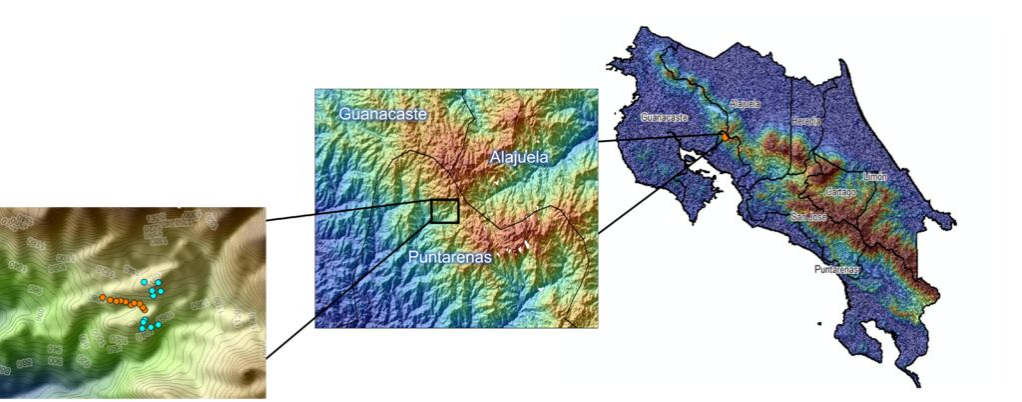
Study areas in the Monteverde region in Costa Rica. Blue dots indicate forest trapping sites, while orange dots indicate open area trapping sites.

At each of the habitat types, traps were established along a transect, with each sub-unit being 100 m apart (Fig. 1). In the two forested transects, five sub-units were equipped with a canopy and understorey trap. Understorey traps were installed at a height of 1.5 m above the ground. In the open area site, ten traps 1.5 m above the ground were established at different sub-units, each 100 m apart. Sampling the canopy in addition to the understorey allowed us to expand the potential biodiversity in our samples, as canopy sampling is rarely included in most Euglossine trapping studies and canopy specialists are potentially under-represented (Sobek et al. 2009, Ulyshen et al. 2010, Urban-Mead et al. 2021).

### Quality control

**Figure 2.**
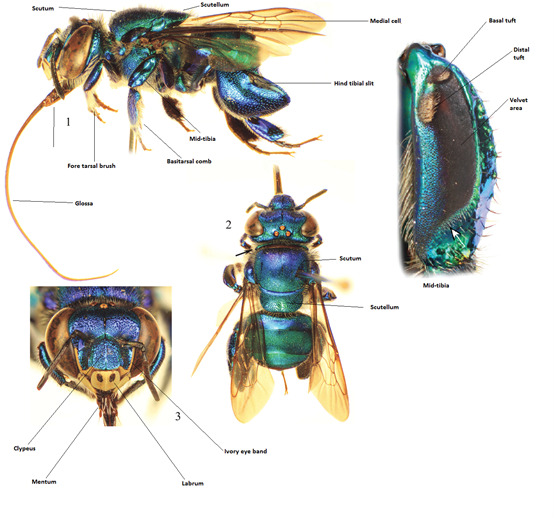
Identifying features of Euglossini (Nemésio and Engel 2012).

Individuals collected at each trap were placed in an 9.58 cm x 17.78 cm Whirlpack plastic pouch. The bags were padded with tissue paper to prevent the movement of live bees from damaging themselves or other specimens. Each collection bag was labelled with the name of the sampling site, date collected, name of the individual collecting the sample and the trap number. The bees were returned to the laboratory and placed in the freezer for at least 24 hours to ensure that all of the specimens were dead before identification. Specimens were mounted or preserved according to standard protocols as follows:

1. The bags were removed from the freezer and each specimen placed in a jar containing 70% ethanol. This is done do remove any residue from the baiting compound or other compounds collected by males from the mid-tibial tuft which could interfere with identification.
2. The bees were removed from the ethanol and placed on a sheet of tissue paper to dry and were identified while still flexible.
3. The bees were mounted, taking into consideration the visibility of the most important diagnostic features of their anatomy. Fig. 2 is an example of a mounted orchid bee. Note that the mid-legs are positioned to ensure that the middle tibia and velvet area are clearly visible and that the forelegs and antenna do not obscure the mandibles or ivory bands, respectively.
4. Mounted specimens were dried at approximately 65^0^C for at least seven days before being placed in long term storage cabinets. This drying period prevents moisture build-up and subsequent moulding of stored specimens.
5. A minimum of 50 individuals per species were mounted and the remaining specimens were preserved in ethanol.

Standard procedures were followed during specimen sorting and identification in the laboratory. All Euglossine specimens were identified to species level using physical characteristics as described in Roubik and Hanson’s dichotomous key in "Orchid bees of tropical America Biology and Field Guide" (Roubik and Hanson 2004). Each specimen was labelled and assigned with a unique specimen code consistent with the collection database. All specimens are deposited in the reference collection at the CIEE campus.

The resulting specimen collection will be used to provide information for future research and as an education tool. Pollinaria attached to bees at the time of collection were preserved on the specimens for future identification. Specimens of non-target taxa were mounted or preserved in ethanol for future identification.

## Geographic coverage

### Description

The study was conducted at the Council on International Educational Exchange (CIEE) Monteverde in Puntarenas Province, San Luis, Monteverde, Costa Rica, formerly known as University of Georgia Costa Rica. This site is positioned on the Pacific slope of the Tilarán Mountain Range and borders two nature preserves. The 62.7 ha campus (centred at 10.2827°N, 84.7985°W) consists of three distinct habitat types: 1) secondary premontane wet forest with 60 years of growth (elevation 1,100-1,170 m); 2) open low-intensity agricultural areas; and 3) areas considered highly modified with roads, multi-use recreation areas and buildings/structures within a 40 m elevation range (Fig. 1). Fifty ha are secondary forest and the remaining 12 ha consist of open pasture or facilities. This area exhibits weather patterns consistent with a Central American premontane wet forest, with the driest months being January-April and the wettest months being May-October. Peak dry season is April, corresponding to the highest average temperatures. October is the month with the most rainfall and the lowest average temperatures (World Weather & Climate Information 2019). The average annual temperatures range between 15.6 and 29.4^0^C. The Monteverde region in Costa Rica is largely covered by montane forest, also classified as cloud forest. The area sampled in this study is premontane wet forest, which has elevation of (1,100-1,500 m) and receives an average of 2.4 m of rainfall annually. Premontane wet forest is slightly lower in elevation than cloud forest.

### Coordinates

10.279°N and 10.285°N Latitude; -84.795°W and -84.807°W Longitude.

## Taxonomic coverage

### Description

**Tribe Euglossini** (Arthropoda: Insecta: Hymenoptera: Apidae: Corbiculata)

Physical descriptions and baseline distribution and elevation data were based on Roubik and Hanson's "Orchid bees of tropical America Biology and Field Guide" (Roubik and Hanson 2004) and cross-referenced with GBIF databases to check for any updates (GBIF.org 2021a, GBIF.org 2021b, GBIF.org 2021c, GBIF.org 2021d, GBIF.org 2021f, GBIF.org 2021g, GBIF.org 2021h, GBIF.org 2021i). Table 3 includes catch numbers of each species. The dataset includes 36 species across four genera of Euglossini (*Eufriesea*, *Euglossa*, *Eulaema* and *Exaerete*) and includes new records for the Monteverde region and expanded elevation range of the following taxa:

GBIF.org 2021a Fuentes 1993

***Eufrieseamussitans* (Fabricius, 1787)**

**Identification.** Body 17-21 mm long. The clypeus has two strong sublateral ridges and a medial ridge. The medial ridge distinguishes *E.mussitans* from *E.concava*, the latter of which exhibits a concave area between the sublateral ridges.

**Remarks**. This species has been documented from “Mexico to south-eastern Brazil; lowlands up to 1,000 m” (Roubik and Hanson 2004). In Costa Rica, *E.mussitans* has been documented in the northern Provinces of Guanacaste and Alajuela (GBIF.org 2021b). This is the first known record of *E.mussitans* in the Puntarenas Province and at the elevation of 1,041-1,168 m. This study documented 1,025 *E.mussitans* individuals.

***Eufriesearufocauda* (Kimsey, 1977)**

**Identification.** Body ~ 14 mm long. Clypeus does not possess sublateral ridges. Face bronze below and green above in males. The anterior part of tergum II is dark and the posterior part of tergum II is reddish-copper with yellow hairs. In lateral view, male labrum has a square outline; when viewed dorsally, a pair of prominent conical points is visible. Differs from *E.chrysopyga* as: 1) *E.chrysopyga* has a uniformly purple tergum II; and 2) lateral view of male labrum of *E.chrysopyga* has a triangular outline and dorsal view reveals barely visible pair of conical points.

**Remarks**: In Costa Rica, *E.rufocauda* has been previously documented in Limon, Alajuela, Heredia and Guanacaste (North and in provinces on the Caribbean side) (GBIF.org 2021c). This study provides occurrence data for *E.rufocauda* in the Monteverde region of the Puntarenas Province and at the elevation range of 1,092-1,154 m. Four *E.rufocauda* individuals were documented.

***Euglossadodsoni* (Moure, 1965)**

**Identification.** Body 10 mm long and reddish-bronze. Green clypeus and complete ivory eye bands. Basal tuft on the middle tibia is inconspicuous, thus appearing to have just one tuft. This distinguishes it clearly from very similar *E.erythrochlora* (not documented in this study), which has two apparent tibial tufts.

**Remarks.** This species has been documented from “Costa Rica to Columbia; lowlands up to at least 800 m” (Roubik and Hanson 2004). *Euglossadodsoni* has been documented in all provinces of Costa Rica, though only in the southern half of the Puntarenas Province (Monteverde is in the northern part of the Province, bordering Alajuela) (GBIF.org 2021d). This study provides expanded occurrence data for *E.dodsoni* in the Monteverde region and at the elevation of 1,041-1,168 m. Fifteen *E.dodsoni* individuals were documented.

***Euglossadressleri* (Moure, 1968)**

**Identification.** Body 12 mm long and light green. Mesosoma is sometimes bronzish. Tongue is much shorter than the body. Clypeus is blue and the labrum has two distinct black spots. Ivory eye bands are absent. Two tufts are present and spaced well apart on the middle tibia, with distal tuft barely notched.

**Remarks.** In Costa Rica, *E.dressleri* has been documented in San Vito in Puntarenas and Peñas Blancas in Alajuela (GBIF.org 2021e). This study expands the known distribution of *E.dressleri* to include Monteverde and at an elevation of 1,157 m. One *E.dressleri* individual was documented.

***Euglossahansoni* (Moure, 1965)**

**Identification.** Body 10 mm long and reddish-bronze or green. Thorax is shiny with shallow and reduced punctures. Tongue approximately the length of the body. Top of the head is predominantly green, sometimes with bronze. Ivory eye bands are complete and clypeus is blue. Middle tibia appears to have three tufts. Similar to *E.alleni* (not documented in this study) and *E.purpurea*, though *E.alenni* is very rare with less punctuation on tergum II and *E.purpurea* is more reddish with blackish clypeus.

**Remarks.** In Costa Rica, *E.hansoni* has been documented in Heredia, Limon and southern Puntarenas in the Osa Peninsula and the coastal nature preserve Refugio National de Fauna Silvestre Golfrio (GBIF.org 2021f). This study provides expanded occurrence data for *E.hansoni* at 1,095-1,157 m and in the region of Monteverde. Seven *E.hansoni* individuals were documented.

***Euglossaignita* (Smith, 1874)**

**Identification.** Body is 14-15 mm long with a green mesosoma and reddish-bronze metasoma. Tongue is longer than the body. Ivory bands are complete and clypeus is green. Broad longitudinal depression present in the middle of the scutellum. Two tufts on the middle tibia are touching, possibly appearing to be one tuft. Distal tuft is larger. A pair of widely separated diagonal slits is present on scutellum II, each with a dense row of setae.

**Remarks.** *Euglossaignita* has been documented in all seven provinces in Costa Rica: Guanacaste, Alajuela, Heredia, Cartago, Limon and southern Puntarenas on the Osa Peninsula (GBIF.org 2021g). This study documents occurrences of *E.ignita* in the Monteverde region and at an elevation of 1,083-1,137 m. Five *E.ignita* individuals were documented.

***Euglossatridentata* (Moure, 1970)**

**Identification.** Body is green and 11-12 mm long; tongue is much shorter than the body. Ivory eye bands are complete and clypeus is green. Three teeth on mandibles. Middle tibia has two tufts with distal tuft having a shallow notch. Similar to *E.deceptrix* and E. *variabilis*, though *E.deceptrix* and *E.variabilis* both have two teeth on the mandibles.

**Remarks.** In Costa Rica, *E.tridentata* has been recorded in Guanacaste, Alajuela, Heredia, Limon, San Jose and Puntarenas, outside of the Monteverde region (GBIF.org 2021h). This study provides expanded occurrence data for *E.tridentata* at 1,041-1,168 m and in the Monteverde region. Seventy-four *E.tridentata* individuals were documented.

***Euglossaturbinifex* (Dressler, 1978)**

**Identification.** Body is 11 mm long and mostly green with a bluish-green mesosoma. Tongue is the length of the body. Mandibles have two teeth. Two tufts on the middle tibia, basal tuft larger than distal one. Sternum II has a pair of small semicircular depressions containing rows of setae. Similar to *E.bursigera*, but *E.bursigera* often has a more bronzish body and has three teeth on the mandible.

**Remarks.** In Costa Rica, *E.turbinifex* has been recorded in Alajuela, Heredia and Limon. In the GBIF database, less than 40 specimens are documented in total, 20 of them from Costa Rica (GBIF.org 2021i). This study documents one occurrence of *E.turbinifex* in the Monteverde region of the Puntarenas Province at an elevation of 1,154 m. One *E.turbinifex* individual was documented.

### Taxa included

**Table:** 

Rank	Scientific Name	
phylum	Arthropoda	
subphylum	Hexapoda	
class	Insecta	
order	Hymenoptera	
superfamily	Apoidea	
family	Apidae	
subfamily	Apinae	
tribe	Euglossini	
genus	*Eufriesea*	
genus	*Euglossa*	
genus	*Eulaema*	
genus	*Exaerete*	

## Temporal coverage

### Notes

9 January 2019 to 4 October 2019

## Usage licence

### Usage licence

Creative Commons Public Domain Waiver (CC-Zero)

## Data resources

### Data package title

Euglossine bee catches of Monteverde, Costa Rica 2019

### Resource link

https://scan-bugs.org/portal/collections/misc/collprofiles.php?collid=299

### Number of data sets

1

### Data set 1.

#### Data set name

Euglossine bee catches of Monteverde, Costa Rica 2019

#### Download URL

https://www.gbif.org/dataset/324ca21e-71f1-4480-a09f-6f6f4a1ad5ba

#### Description

This dataset contains 2,742 entries of Euglossine bees across four genera in Monteverde, Costa Rica (McDonald et al. 2021).

**Data set 1.** 

Column label	Column description
institutionID	Identifier for the institution where database originates.
collectionCode	Prefix of specimen code.
catalogNumber	Full specinen code including collection code ("UGAEug" = UGA Euglossine) and specimen number in the collection.
month	Month specimen was collected.
verbatimEventDate	Date specimen was collected.
country	The name of the country in which the Location occurs (Costa Rica).
countryCode	ISO code of the country in which the location occurs.
scientificName	Scientific name of specimen.
sex	Sex of specimen (all male in this dataset).
basisOfRecord	Specific nature of the data record.
locality	Location of trap sites (University of Georgia Costa Rica campus).
habitat	Habitat type (open area or forested area) in which specimen was caught.
occurrenceRemarks	Vertical position of trap in which specimen was caught. Trap sites in forested areas possessed traps in both the canopy and understorey, whereas open area traps were only placed in one vertical position near the ground. Traps in open areas are labelled "open", as their vertical position was static.
fieldNumber	Alphanumeric trap location code consisting of one letter ("F" or "O") and a number 1-10 (F = forest, O = open)
decimalLongitude	Longitude of trap site
decimalLatitude	Latitude of trap site
geodeticDatum	The set of reference points on the Earth's surface upon which the geographic coordinates in "decimalLatitude" and "decimalLongitude" are based (WGS84, a constant).
minimumElevationInMetres	Elevation (in metres) of trap site.
recordedBy	Individual who collected specimen.
coordinateUncertaintyInMetres	Uncertainty of the coordinates of the centre of the sampling area.
rightsHolder	The organisation owning the rights over this resource (for this dataset, University of Georgia).
class	Class name.
collectionID	Identifier for the collection publishing the data.
day	Day specimen was collected.
year	Year sepcimen was collected.
kingdom	Kingdom name.
phylum	Phylum name.
class	Class name.
order	Order name.
family	Family name.
genus	Genus name.
scientificNameAuthorship	Name of the author of the lowest taxon rank in the record.
specificEpithet	Species epithet of the scientific name.
day	Day the specimen was collected.
year	Year in which the specimen was collected.
accessRights	Information regarding who can access this information and use restrictions.
taxonRank	The lowest taxonomic rank of the record.
eventDate	Date the specimen was collected.
occurrenceID	A global unique identifier for the occurrence.
preparations	Preservation method of the specimen.
startDayOfYear	Integer day on which specimen was collected.
taxonID	Identifier for the set of taxon information (global identifier in this dataset).
references	Reference page link for occurrence.
modified	Date and time of last update.
id	A unique identifier for the Symbiota Collection of Arthropods Network (SCAN). Equivalent here to eventID.
preparations	Method of specimen preservation (mounted, in ethanol or frozen).
recordID	A Universally Unique IDentifier (UUID) used to uniquely identify an object published to the internet, in this case an occurrence record.

## Additional information

Due to changing climatic conditions and other anthropogenic effects, consistent sampling at various elevations is needed to track distributional changes in fauna over time. Shifting ranges are predicted as Euglossine bees respond to climate change (Silva et al. 2015) and, thus, a complete understanding of their current range will be critical for tracking future shifts. Given the co-dependent and intimate relationships between orchids and Eulossine bees, it is important to monitor populations and species of both the groups to detect future declines or recovery under restoration practices. Our 10-month study sampled across wet and dry seasons, in both forest and open areas, as well as canopy and understorey and was, therefore, able to yield a more complete species-list in this region, including records of highly seasonal species such as *Eufrieseamussitans*. This demonstrates that a 12-month study would be useful in detecting other ephemeral species that occur in November and December and we suggest that continued monitoring of orchid bees is needed for their protection and conservation efforts in such hyper-diverse and endemic tropical ecosystems.

## Figures and Tables

**Table 1.** List of trap stations, elevation and location in Monteverde, Costa Rica. F = Forest traps, O = open area traps.

**Trap Station**	**Elevation (m)**	**Latitude / Longitude**
F1	1158	10°17'4.776", -84°47’59.9994"
F2	1168	10°17'4.632", -84°47'54.6"
F3	1154	10°17'1.6794", -84°47'57.1194"
F4	1121	10°16'59.8074", -84°47'56.4"
F5	1137	10°17'1.5354", -84°47'53.8794"
F6	1095	10°16'51.132", -84°47'59.6394"
F7	1092	10°16'50.3394", -84°47'59.9994"
F8	1083	10°16'48.036", -84°48'0.36"
F9	1055	10°16'48.396", -84°47'57.48"
F10	1041	10°16'49.4754", -84°47'54.6"
O1	1084	10°16'54.6702", -84°47'59.6394"
O2	1081	10°16'55.7112", -84°48'0.36"
O3	1085	10°16'56.9634", -84°48'1.4394"
O4	1084	10°16'57.2664", -84°48'3.2394"
O5	1080	10°16'56.6358", -84°48'4.6794"
O6	1094	10°16'57.6726", -84°48'6.48"
O7	1096	10°16'58.0368", -84°48'8.28"
O8	1093	10°16'57.9396", -84°48'10.08"
O9	1092	10°16'58.3638", -84°48'12.2394"
O10	1083	10°16'59.1666", -84°48'14.76"

**Table 2.** Sampling dates and habitat types sampled during each month of the study in 2019 in Monteverde, Costa Rica.

**Month**	**Sampling Dates**	**Areas Sampled**
January	3-7 January 2019	Forest A
February	6-10 February 2019	Forest A
March	9-13 March 2019	Forest A
April	9-13 April 2019	Forest A; Forest B
May	30 April - 4 May 2019	Forest A
June	26-30 May 2019	Forest A
June	17-21 June 2019	Forest A; Open
July	1-5 July 2019	Forest A; Open
August	1-5 August 2019	Forest A; Open
September	1-5 September 2019	Forest A; Open
October	30 September- 4 October 2019	Forest A; Forest B; Open

**Table 3.** List of Euglossine species including number of trapped male adults.

**Bee Species**	**Number of Adult Males**
*Eufrieseachrysopyga*	10
*Eufrieseaconcava*	123
*Eufrieseamacroglossa*	33
*Eufrieseamussitans*	1,025
*Eufriesearufocauda*	4
*Eufrieseaschmidtiana*	2
*Euglossaallosticta*	43
*Euglossabursigera*	1
*Euglossachampioni*	21
*Euglossacybelia*	21
*Euglossadeceptrix*	5
*Euglossadilemma*	227
*Euglossadissimula*	1
*Euglossadodsoni*	15
*Euglossadressleri*	1
*Euglossagorgonensis*	5
*Euglossahansoni*	7
*Euglossaheterosticta*	6
*Euglossaignita*	5
*Euglossaimperialis*	898
*Euglossamacroglossa*	2
*Euglossamaculilabris*	91
*Euglossamixta*	2
*Euglossapurpurea*	2
*Euglossatridentata*	74
*Euglossaturbinifex*	1
*Euglossavariabilis*	29
*Euglossavillosa*	1
*Eulaemabombiformis*	4
*Eulaemameriana*	8
*Eulaemanigrita*	6
*Eulaemapolychroma*	7
*Eulaemaseabrai*	2
*Exaeratesmaragdina*	1
*Exaeretefrontalis*	58
*Exaeretesmaragdina*	1
